# No‐reflow after stroke reperfusion therapy: An emerging phenomenon to be explored

**DOI:** 10.1111/cns.14631

**Published:** 2024-02-15

**Authors:** Milan Jia, Feiyang Jin, Sijie Li, Changhong Ren, Mangal Ruchi, Yuchuan Ding, Wenbo Zhao, Xunming Ji

**Affiliations:** ^1^ Department of Neurology, Xuanwu Hospital Capital Medical University Beijing China; ^2^ Department of Emergency, Xuanwu Hospital Capital Medical University Beijing China; ^3^ Beijing Key Laboratory of Hypoxic Conditioning Translational Medicine, Xuanwu Hospital Capital Medical University Beijing China; ^4^ Department of Neurosurgery Wayne State University School of Medicine Detroit Michigan USA; ^5^ Department of Neurosurgery, Xuanwu Hospital Capital Medical University Beijing China

**Keywords:** acute ischemic stroke, endovascular thrombectomy, microvascular disturbance, no‐reflow phenomenon, reperfusion therapy

## Abstract

In the field of stroke thrombectomy, ineffective clinical and angiographic reperfusion after successful recanalization has drawn attention. Partial or complete microcirculatory reperfusion failure after the achievement of full patency of a former obstructed large vessel, known as the “no‐reflow phenomenon” or “microvascular obstruction,” was first reported in the 1960s and was later detected in both experimental models and patients with stroke. The no‐reflow phenomenon (NRP) was reported to result from intraluminal occlusions formed by blood components and extraluminal constriction exerted by the surrounding structures of the vessel wall. More recently, an emerging number of clinical studies have estimated the prevalence of the NRP in stroke patients following reperfusion therapy, ranging from 3.3% to 63% depending on its evaluation methods or study population. Studies also demonstrated its detrimental effects on infarction progress and neurological outcomes. In this review, we discuss the research advances, underlying pathogenesis, diagnostic techniques, and management approaches concerning the no‐reflow phenomenon in the stroke population to provide a comprehensive understanding of this phenomenon and offer references for future investigations.

## Graphical abstract

The illustration of the consequences, pathogenesis, diagnostic methods, and promising management of the cerebral no‐reflow phenomenon.

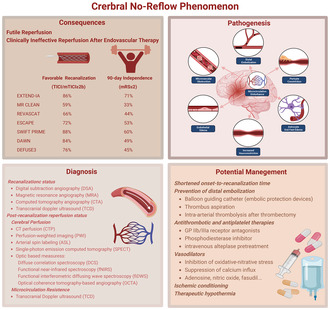

## 1 INTRODUCTION

Acute ischemic stroke (AIS), especially in patients with intracranial proximal large vessel occlusion, is a major cause of mortality and morbidity worldwide.^1^ Timely recanalization with intravenous thrombolysis and endovascular thrombectomy is currently the first choice for AIS management and has effectively improved the clinical outcomes of stroke patients.^2^ However, there remains a gap between successful recanalization and a good prognosis, as almost half of the patients do not experience favorable outcomes despite successful thrombectomy and the full patency of the occluded artery being achieved.^3^ This discrepancy may be ascribed to many reasons, including established infarction preceding the recanalization, premorbid poor collateral reserve, comorbidities, and complications. It is significant to note that successful recanalization might not be equivalent to successful reperfusion, as recent studies have found that over 30% of patients who achieved a modified Thrombolysis in Cerebral Ischemia (mTICI) score of 3 after thrombectomy, which in general, is considered as complete patency of the affected vessel, still suffered from sustained hypoperfusion in certain areas.^4^, ^5^ Such perfusion deficiency after successful recanalization is known as the no‐reflow phenomenon (NRP), the clinical manifestation of the functional and structural alterations in the microcirculation during the ischemia–reperfusion process.^6^ It is worth noting that the concept of NRP is not identical to concepts like Futile Reperfusion (FR)^7^ and Clinical Ineffective Reperfusion (CIR),^8^ which are more extensively used and indicate unfavorable functional outcomes despite successful recanalization whether under guideline‐recommended management or not. The NRP specifically refers to the microcirculation reperfusion failure despite recanalization of the occluded large artery, which offers insights into the understanding of possible courses of both FR and CIR. To date, the NRP has been detected in various circulatory systems, including the heart, kidney, and skeletal muscle. Inspiringly, research has developed rapidly regarding the risk factor screening, diagnosis, and management of the NRP in coronary diseases.^9^ However, not as much has been realized in stroke research, despite the fact that this phenomenon was first detected in the brain rather than in the heart. Therefore, this review will briefly introduce the research history of the cerebral NRP, summarize its underlying pathogenesis, and provide a contemporary overview of its diagnostic and management strategies to offer a deeper insight into this field and advance future studies.

## 2 HISTORY OF NRP RESEARCH

In 1968, the NRP was first described by Ames et al.^6^ in a rabbit model. In their experiment, with prolongation of ischemia to 5 or 7.5 min, cerebral flow failed to be fully restored after relief of the vessel obstruction due to the blockage of capillaries by erythrocytes. Following this, microcirculation disturbance, with reperfusion deficiency after embolization removal, was discovered in other animal models, ranging from total to focal cerebral ischemia.^10^, ^11^, ^12^ Ames et al. speculated that impaired microcirculation could be attributed to changes in the blood components, vascular lumen, or both. This conjecture was later confirmed. Under the microscope and in vivo two‐photon imaging, it was directly observed that the vascular lumen could be blocked by assorted blood components such as platelets, fibrin, and blood cells.^13^, ^14^, ^15^, ^16^, ^17^ Moreover, exterior compression by compositions of the capillary wall and the surrounding structure also deteriorates lumen constriction.^12^, ^18^, ^19^

Early research efforts were directed toward the exploration of potential pathophysiological mechanisms, while the incidence and clinical impact of the NRP in stroke patients were not investigated as thoroughly, due to the unavailability of effective revascularization strategies along with the restriction of imaging assessment techniques in clinical settings.^20^, ^21^ It was not until 1994, with the ubiquity of thrombolytic agents, that the NRP was detected in the human brain for the first time. Through single‐photon emission computed tomography (SPECT), researchers noted a mismatch between arterial recanalization and successful reperfusion in a stroke patient receiving streptokinase thrombolysis.^22^

Later in the 2010s, the full patency of the obstructed artery could be realized and validated under angiography.^23^, ^24^ Some studies discovered that the reperfusion state is superior to the recanalization state in terms of prognosis prediction, indicating the significance of the post‐recanalization reperfusion failure.^4^, ^25^, ^26^ Such a condition has once again set off an upsurge in the NRP research. Recent studies have focused on the investigation of the incidence, prognostic value, correlative risk factors, and management of the NRP in stroke patients who underwent recanalization therapy.^5^, ^27^

## 3 PATHOPHYSIOLOGICAL PERSPECTIVE

A thorough interpretation of the pathophysiological mechanisms of the NRP may help to develop evaluation techniques and effective management. In 1968, Ames et al.^6^ first demonstrated the entrapped erythrocytes at the capillary level after recanalization, marking the first identification of the NRP. Later, under electron microscopy and erythrocyte autofluorescence, such a phenomenon was detected in other studies in both the ischemic core and the penumbra.^12^, ^28^, ^29^

**FIGURE 1:**
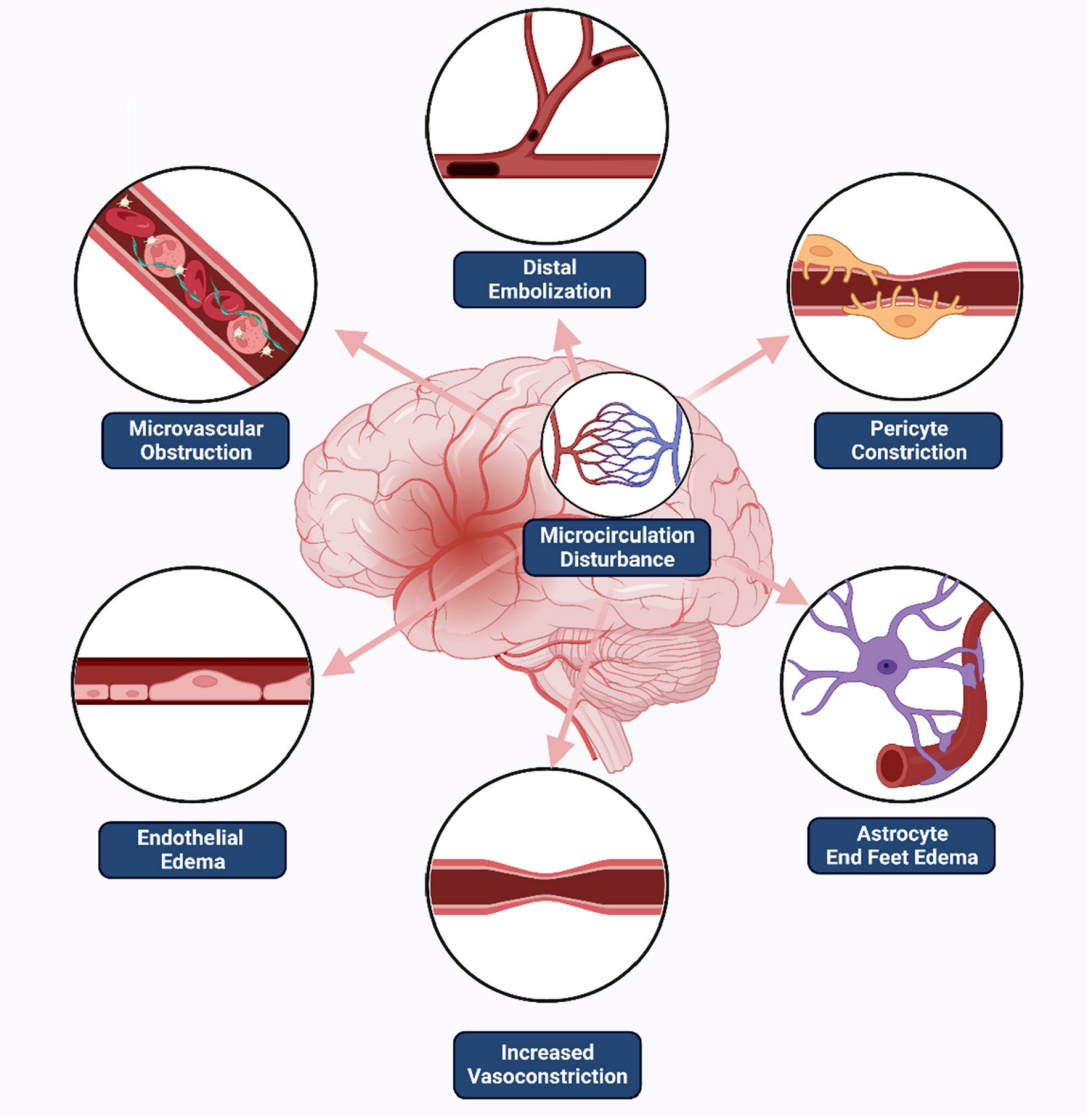
Illustration of the no‐reflow phenomenon pathogenesis (Created with BioRender.com) The no‐reflow phenomenon, which refers to the post‐recanalization reperfusion failure, is the clinical manifestation of microcirculation disturbance. The latter is primarily attributed to mechanical obstruction by blood components or clog fragmentation, and compression due to functional or structural change in the vessel wall or the surrounding cells.

Microcirculatory disturbance related to the NRP may lead to both inadequate supply of the peripheral energy substrate and insufficient delivery of metabolites. Continuous hypoperfusion in the penumbra may lead to infarction expansion and further neurological impairment despite successful recanalization success.^30^ Alternatively, persistent hypoperfusion in the ischemic core might potentially affect the resolution of edema, the clearance of necrotic tissues, and collateral angiogenesis, which would ultimately impact neural plasticity and tissue repair. While it is clear that the abnormal vascular events inducing the NRP occur at the microvasculature level, the underlying mechanisms still remain unclear.^31^ According to current research, the NRP is primarily attributed to mechanical obstruction by blood components or clog fragmentation, and compression due to functional or structural change in the vessel wall or the surrounding cells (Figure 1).

### 3.1 Potential mechanisms accounting for the obstruction of the microcirculation

The blockage of the microcirculation may be secondary to distal embolization from the proximal cerebral arteries, in‐situ thrombosis, and microcirculatory stasis. During reperfusion therapy, a thrombus may arise from clot fragmentation, which could drift to distal vasculature, resulting in incomplete reperfusion after recanalization.^32^ Recently, via high‐resolution MRI, studies have discovered the existence of peripheral emboli within the vascular territory after digital subtraction angiography (DSA)‐validated full patency of proximal large vessels.^33^ This validated the role of clot fragments in microvascular circulation disturbance. Considering the amount and location of the fragments, it remains uncertain how such thrombus migration would influence the outcome of successful recanalization.^34^ Additionally, the fracture properties and migration tendency may vary among clots of different sizes and compositions or diverse collateral conditions.^35^, ^36^

During an ischemic attack, the narrowed microvascular lumina may be clogged with platelets, leukocytes, and fibrin, which also contributes to hypoperfusion after vessel recanalization. Platelets play a pivotal role in thrombogenesis and microcirculation blockage after stroke. Thrombi that were abundant in platelets and erythrocytes were observed in the capillaries and precapillary arterioles in a baboon model of 3‐hour middle cerebral artery occlusion (MCAO) and blood flow restoration.^17^ In addition to being involved in micro‐thrombi formation, platelets may also be implicated in the NRP, serving as a “bridge” in multifaceted interactions between cells and factors within the vascular system (e.g., neutrophils, P‐selectin), along with the neighboring capillary wall elements (e.g., pericytes, endothelium).^37^, ^38^ By releasing mediators and granules, activated platelets enhance the above interactions and further promote lesion progress. Consequently, antiplatelet treatment, which includes preventing platelet activation, adhesion, or aggregation, along with therapy that inhibits interactions between platelets and other elements, could improve blood flow after reperfusion in animal models.^39^

Fibrin deposition, along with the activation of tissue factor‐mediated coagulation, is also involved in the impairment of microvascular patency. Okada et al.^40^ found that in a primate reversible MCAO model, fibrin deposition in the microvasculature accumulated in a time‐dependent manner. More recently, via near‐infrared fluorescence (NIRF) with an FXIIIa‐targeted probe and histopathological analysis, Chen et al.^41^ confirmed the existence of fibrin deposition and erythrocyte aggregation in the capillaries of mice after thrombolysis. Thomas et al.^15^ detected an increase of reflow in micro‐vessels of all sizes after the inhibition of coagulation via the murine anti‐tissue factor monoclonal antibody TF9‐6B4, suggesting the potential effect of tissue factor‐mediated coagulation in microvascular perfusion deficit. The suppression of fibrinolysis, on the other hand, through the upregulation of the type 1 plasminogen activator inhibitor (PAI‐1) gene, may aggravate the fibrin deposition and further impair the microcirculation.^42^

Another proposed mechanism of the NRP is capillary flow stagnation due to polymorphonuclear leukocyte blocking inside the lumen. Early in 1989, Grogaard et al.^43^ discovered that intraperitoneal injections of an antineutrophil serum extracted from sheep could facilitate the reperfusion status in a rat model, suggesting the potential role of leukocytes in the NRP. The presence of polymorphonuclear leukocytes was then directly observed in occluded capillaries of baboons by light microscopy.^11^ Two subsequent studies found leukocyte trapping in the microcirculation in MCAO models with early reperfusion.^44^, ^45^ Through two‐photon imaging, investigators directly observed that neutrophils plugged the capillaries in vivo after thrombolysis even with complete clot dissolution, while neutrophil depletion induced by the targeted antibody could facilitate capillary reperfusion and stroke recovery.^29^ Different from permanent cellular plugs involved in no‐reflow, Erdener et.al discovered that neutrophils could stall in a more dynamic way in which they briefly and repetitively got stuck and released.^46^ By blocking polymorphonuclear leukocyte adherence to the microvascular endothelium through anti‐inflammatory approaches, the NRP was seen to be suppressed post‐focal cerebral ischemia.^31^

More recently, Strinitz et al.^47^ collected blood samples from the occluded anterior circulation of stroke patients and detected that there was local neutrophil‐dominant immune cell recruitment, suggesting that there may be potential hazards of microvascular plugging and elevation of blood viscosity. This, in turn, may influence the retrograde collateral flow by increasing vascular resistance.^47^, ^48^ Another form of neutrophil involvement was neutrophil extracellular traps (NETs), a web‐like structure containing DNA released by a neutrophil. These traps play a pivotal role in thrombosis. Emerging evidence suggested the intraluminal occurrence of NETs after prolonged ischemia and the potential impact they may have on the microcirculation.^49^, ^50^

### 3.2 Factors affecting the diameter of the microcirculatory lumen

Cerebral capillary walls consist of endothelial cells, pericytes, basal lamina, and astrocyte end feet, which are distributed in a close alignment. Alternations in the microvascular and perivascular structures, such as swelling or constriction of the neighboring cells, can narrow the lumen, increase vascular resistance, and result in incomplete restoration of blood flow.

Pericytes are present at the outer surface of capillaries and the smallest venules. They are elongated cells, whose long cytoplasmic processes are wrapped around the endothelium. As a crucial component of the neurovascular unit, pericytes exert great influence on blood–brain barrier stabilization, blood flow regulation, immunomodulation, angiogenesis, and neurogenesis, thus providing support for other cellular components and maintaining the normal physiological function of the neurovascular unit.^51^, ^52^ The cytoplasm of pericytes contains actin, myosin, tropomyosin, and desmin, suggesting their capacity for contractile activity. Normally, contractile pericytes can modulate cerebral capillary resistance.^53^ However, during ischemia or even after successful recanalization, pericytes might persistently contract due to multifaceted pathways including intracellular calcium overload, excitotoxicity, and oxidative‐nitrative stress.^19^, ^53^, ^54^, ^55^ Such contraction, along with subsequent pericyte death in rigor mortis, would cause long‐lasting capillary constriction and incomplete microvascular blood restoration, leading to limited oxygen supply and subsequent infarction progress with time.^19^, ^56^ On the contrary, one study dissented from the contribution of pericytes in microcirculation regulation. Instead, this study found arteriolar smooth muscle cells as the mediator of the capillary blood flow under both physiological and pathological conditions.^57^

Increased vasoconstriction of parenchymal arterioles (PAs), in response to ischemia and reperfusion, also contributes to the microvascular perfusion deficit. PAs are long and relatively unbranched vessels connecting the pial vessels to the capillaries, and they have been shown to bottleneck the flow within the cortex due to their high resistance. During postischemic reperfusion, PAs may remain constricted. This influences the basal tone or myogenic reactivity and limits focal blood restoration in the ischemic region.^18^, ^58^

Functional and structural changes of other elements in the neurovascular unit have also been found to aggravate a narrowing lumen. Early in 1964, Hills et al.^59^ reported astrocytic swelling in ischemic rat models but overlooked its potent impingement on the capillary lumen and perfusion. It was later found that the swelling of the perivascular astrocyte end‐feet would compress the capillaries.^12^, ^20^ Investigators additionally detected that the paucity of oxygen and substrates, due to blood flow interference, may lead to energy failure of the cellular sodium‐potassium pump and electrolyte disturbances, which are involved in the swelling of endothelial cells and astrocytes.^60^ Furthermore, cerebral edema was also observed due to the breakdown of the blood–brain barrier and cerebrospinal fluid influx.^61^ In conclusion, both cellular and perivascular swelling could lead to capillary compression, impair the patency of the micro‐vessels, and thus contribute to the NRP.

## 4 EVALUATION OF THE NRP

Despite abundant imaging and histological analytical methods available in animal models for direct visualization of microcirculation, practical challenges still exist over microcirculatory perfusion status assessment in stroke patients. Therefore, there are limited trials concerning the NRP prevalence in stroke patients with wide variation from 0% to 81% (as shown in Table 1).^26^, ^27^, ^62^ This variation may be attributed to the difference in evaluation techniques and definitions of the NRP across studies, as some studies set a specific cutoff value to define reperfusion deficiency, while others consider a relative change from the baseline or even surrogate indicators of microcirculatory resistance for the NRP diagnosis.^63^, ^64^, ^65^, ^66^ At present, no consensus has been reached neither on the definition and diagnostic criteria for the NRP nor the optimal choice of diagnostic imaging techniques. Thus, we will discuss the relative merits and shortcomings of current imaging approaches with regard to the stroke population.

**TABLE 1:** Previous studies investigating NRP in stroke patients.

First author, year	Treatment	Recanalization				Reperfusion			Number of recanalized patients with hypoperfusion
		OTR	Image	Assessment time	Definition of recanalization	Image	Assessment time	Definition of hypoperfusion	
Baird, 1994^22^	IA SK	4–24 h	DSA	24 h	Partial or complete	SPECT	24 h	Perfusion level ≤12% of the homologous region	1/4 (25%)
Yasaka, 1998^67^	IV SK or placebo	<4 h	TCD	24 h	Partial or complete	SPECT	24 h	Perfusion level ≤12% of the homologous region	4/8 (50%)
Khatri, 2005^62^	IV + IA t‐PA	<3 h	DSA	On DSA completion	Complete (AOL III)	DSA	On DSA completion	TIMI 0–2	26/32 (81%)
Albers, 2006^77^	IV t‐PA	4–24 h	MRA	3–6 h after t‐PA	Partial or complete	PWI	3‐6 h after t‐PA	Tmax ≥2 s	4/19 (21%)
De Silva, 2009^63^	IV t‐PA	3‐6 h	MRA	3–5 days	Partial or complete (TIMI 2–3)	PWI	3–5 days	Perfusion lesion (Tmax ≥2 s) Reduction ≤90%	4/13 (31%)
Soares, 2010^78^	IV t‐PA ± MT or no treatment	<6 h	CTA	24 h	Partial or complete (Recanalization Index >50%)	CTP	24 h	CBV <2.0 mL/100 g, CBF <66%, MTT >145%	5/13 (38%)
Bivard, 2013^69^	IV t‐PA or no treatment	<6 h	MRA	24 h	Complete (TIMI3)	ASL	24 h	Mean ± 2SD of normal pixels from the healthy hemisphere	0%
Eilaghi, 2013^79^	IV t‐PA or no treatment	<4.5 h	CTA	≤24 h	Partial or complete (TIMI 2–3)	CTP	≤24 h	Absolute Tmax reperfusion index ≤58.7%	14/58 (24%)
Marks, 2014^64^	MT ± t‐PA	<12 h	DSA	On DSA completion	Complete (TICI 2b‐3)	PWI	Within 12 h after the procedure	Perfusion lesion (Tmax>6 s) Reduction ≤50%	7/47 (15%)
Horsch, 2015^80^	IV t‐PA or no treatment	<9 h	CTA	3 days	Complete	CTP	3 days	MTT >145%	33 (40%)
Cho, 2015^26^	IV t‐PA or no treatment	<6 h	MRA	<6 h after stroke onset	Partial or complete (AOL II‐III)	PWI	<6 h after onset	Reperfusion ratio <50%	0/13 (0%)
Carbone, 2019^81^	IV t‐PA ± MT or no treatment	<8 h	CTA	24 h	Partial or complete (TIMI 2–3)	CTP	24 h	Reduction of baseline MTT lesion (MTT>145%) ≤75%	15/39 (38%)
Rubiera, 2020^4^	MT ± t‐PA	287 min (177–492)	DSA	On DSA completion	Complete (mTICI 2b‐3)	CTP	≤30 min after recanalization	Tmax ≥6 s	2b: 29/46 (63%) 3: 40/94 (42.5%)
Schiphorst, 2021^27^	MT ± t‐PA	<24 h	DSA	On DSA completion	Complete (mTICI 2c‐3)	ASL	24 h	Mean CBF Reduction ≥40%	1/33 (3.3%)
Luby, 2021^82^	MT ± t‐PA	≤12 h	DSA	On DSA completion	Complete (mTICI3)	PWI	24 h	Tmax volume >10 mL with 6 s delay	2/33 (6.67%)
Ng FC, 2022^5^	MT ± t‐PA/Tenecteplase	≤4.5 h	DSA	24 h	Complete (eTICI 2c‐3)	CTP or PWI	24 h	rCBF or rCBV reduction >15%	33/130 (25.3%)

Abbreviations: AOL, arterial occlusion level; ASL, arterial spin labeling; CBF, cerebral blood flow; CBV, cerebral blood volume; CTA, computed tomography angiography; CTP, computed tomography perfusion; DSA, digital subtraction angiography; eTICI, expanded thrombolysis in cerebral infarction; IA, intraarterial; IV, intravenous; MRA, magnetic resonance angiography; MT, mechanical thrombectomy; mTICI, modified thrombolysis in cerebral infarction; MTT, mean transit time; OTT, onset‐to‐recanalization delay; PWI, perfusion‐weighted imaging; rCBF, regional CBF; rCBV, regional CBV; SD, standard deviation; SK, streptokinase; SPECT, single‐photon emission computed tomography; TCD, transcranial Doppler; TICI, thrombolysis in cerebral infarction; TIMI, thrombolysis in myocardial infarction; Tmax, time to max; t‐PA, tissue plasminogen activator.

In early studies, non‐invasive methods, including magnetic resonance angiography (MRA), computed tomography angiography (CTA), and transcranial Doppler ultrasound (TCD), were used to validate successful recanalization in patients receiving intravenous thrombolysis (IV), which only allow evaluation for proximal and relatively large arteries and may cause the overlook of evident distal occlusions.^63^, ^67^ Later in the era of thrombectomy, evaluation by digital subtraction angiography (DSA), performed instantly after EVT, enables direct observation of both macrovascular patency and dynamic collateral perfusion process with excellent spatial and temporal resolution. Grading scales such as the Thrombolysis in Myocardial Infarction (TIMI), Thrombolysis in Cerebral Infarction (TICI), modified TICI (mTICI), extended modified TICI scale (eTICI), and Arterial Occlusive Lesion (AOL) are applied for assessment.^68^ Of note, different definitions of successful recanalization according to these grading scales may influence the estimated frequency of the NRP, as a rigorous standard (like TIMI 3) may lead to a potential risk of the underestimation of the prevalence,^69^ while a moderate inclusion criterion (TICI2b‐2c) may include perfusion abnormalities related to persistent vessel occlusion rather than the microcirculatory disturbance and lead to the NRP overestimation. More recently, Jayme C. Kosior et al. demonstrated the feasibility and potential clinical utility of DSA perfusion (DSAP) which derives a quantitative assessment of perfusion status from standard DSA source images according to conventional contrast bolus‐tracking methodology.^66^ However, it is of note that angiography‐based techniques are performed right after revascularization when patients are still in the catheterization room, thus it might be too early to detect the consistent hypoperfusion and fail to find the true frequency of NRP. Angiography also has limitations in routine evaluation due to its invasive nature and the potential risk of contrast agents.

Other non‐invasive imaging techniques also help to characterize the presence, extent, and localization of the NRP. In the early days, nuclear imaging approaches were applied to detect the NRP in stroke, which have limited use later due to the cost and radiation exposure, especially with the emergence and widespread use of other imaging techniques with high sensitivity and high resolution.^22^, ^67^ Parameters calculated from perfusion imaging like perfusion‐weighted imaging (PWI), diffusion‐weighted imaging (DWI), and computed tomographic perfusion (CTP), give a comprehensive and detailed description of perfusion mappings qualitatively and quantitatively, including time‐to‐maximum of the tissue residue function (Tmax), cerebral blood flow (rCBF), cerebral blood volume (rCBV), time to peak (TTP), or mean transit time (MTT). These perfusion imaging techniques are suitable for routine perfusion evaluation and detection of the NRP, while contrast agent injury and CT‐related radiation injury should be considered during utilization. Other studies implied arterial spin labeling (ASL) for perfusion assessment. ASL provides absolute quantification of CBF with high spatial and temporal resolution and is free of contrast medium injury while may endure the risk of motion artifacts.^27^, ^69^

Moreover, ultrasound‐based methods, such as transcranial Doppler (TCD), allow real‐time bedside monitoring of cerebral hemodynamic status with parameters, such as pulsatility index and resistance index,^70^, ^71^ which is especially suitable for patients with transportation inconvenience or limitation. TCD combined with servo‐controlled finger photoplethysmography (Finapres) enables the assessment of cerebral autoregulation (CA), which refers to the inherent properties of the cerebral vasculature to maintain relatively constant cerebral perfusion in response to rapid fluctuations in cerebral perfusion pressure.^72^ However, TCD measurement refines to large supplying arteries (usually the MCA) while not available for measurement for flow at the brain tissue level. Whether TCD‐related parameters can reflect the perfusion status in microcirculation remains further discussion. More recently, optic‐based techniques, such as diffuse correlation spectroscopy (DCS), functional near‐infrared spectroscopy (fNIRS), functional interferometric diffusing wave spectroscopy (fiDWS), and optical coherence tomography‐based angiography (OCTA), are emerging tools for the dynamic detection of cerebral perfusion status, which are promising for the NRP assessment.^73^, ^74^, ^75^, ^76^ Meanwhile, it should be noted that either optic‐based or ultrasound‐based techniques only characterize perfusion status through surrogate indicators of hemodynamic changes instead of direct visualization and quantitation of microcirculation. Besides, such assessment is influenced by multiple variables such as caffeine intake, room temperature, cognitive activation, mental stress, etc., which brings challenges to the consistency of the experiment environment to ascertain the stability of parameter measurement.^72^ Therefore, results from these approaches should be considered cautiously during interpretation.

In conclusion, the imaging techniques have their own pros and cons with regard to the specific stroke population and application scenario, it is far from certain which approach might be the best. Therefore, a multi‐phase and multi‐technique measurement of the NRP based on individual conditions, disease stage, and application scenario might be preferable for comprehensive NRP evaluation in future clinical settings.

## 5 MANAGEMENT OF THE NO‐REFLOW PHENOMENON

**FIGURE 2:**
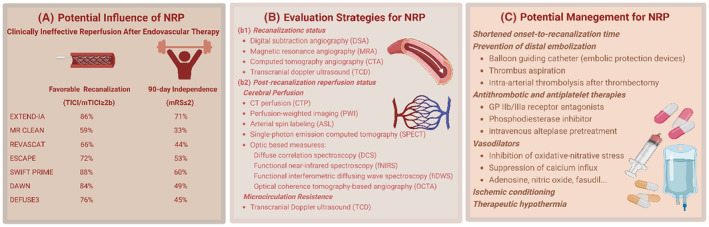
The potential influence, evaluation strategies, and management of the no‐reflow phenomenon in clinical settings (Created with BioRender.com). (A) The potential influence of the NRP. The NRP is involved in clinically ineffective reperfusion after endovascular therapy. The diagram shows favorable recanalization rates (TICI/mTICI ≥2b) and favorable 90‐day clinical outcome proportions (mRS ≤2) in major studies concerning the efficacy of endovascular thrombectomy. It is of note that nearly half of the patients failed to achieve favorable outcomes despite successful recanalization. (B) Evaluation strategies for the NRP. There are currently no available techniques in clinical settings for the monitoring of microcirculation disturbance. After successful recanalization of the proximal large artery based on imaging assessment (b1), strategies for evaluation of cerebral blood flow status or microcirculatory resistance (b2) are applied for detection of the NRP. (C) Potential management for the NRP. Management of the NRP should be emphasized to further improve the functional outcomes following recanalization therapy. Strategies including first‐aid workflow refinement, distal embolization prevention, antiplatelet and antithrombotic therapy, vasodilator, ischemic conditioning, and therapeutic hypothermia, are promising for NRP management.

Clinical management of the cerebral NRP is still insufficient. However, many possibilities are currently being explored to reduce the incidence and extent of NRP in stroke patients by referring to experiences in preclinical studies and cardiovascular research (Figure 2).

### 5.1 Shortened onset‐to‐recanalization time

In animal models, prolonged ischemia was associated with the development of the NRP.^6^ In patients with acute myocardial infarction, a shortened door‐to‐balloon time is associated with a lower incidence of no‐reflow.^83^ Therefore, efforts should be made to optimize the first‐aid workflow and shorten the onset‐to‐recanalization time in stroke patients. In the meantime, the benefit of any strategy for NRP prevention should be weighed up against the time delay in applying it.

### 5.2 Prevention of distal embolization

Strategies like embolic protection devices and thrombus aspiration may reduce the occurrence of clot fragments and thus result in better reperfusion and clinical outcomes, as has been demonstrated in patients who underwent percutaneous coronary intervention (PCI).^84^ Intra‐arterial thrombolysis during or after thrombectomy was demonstrated to improve angiographic reperfusion.^85^

### 5.3 Antithrombotic and antiplatelet therapies

Antiplatelet and thrombolytic therapies may prevent the NRP. In animal studies, GP IIb/IIIa receptor antagonists could effectively block fibrinogen‐dependent platelet aggregation, thereby preventing cerebral microvascular thrombosis.^37^, ^39^, ^86^, ^87^ It was also preliminarily observed in some trials that tirofiban, either through oral, intravenous, or intra‐arterial administration, may improve functional outcomes and reduce mortality in AIS patients undergoing reperfusion therapy.^88^, ^89^ Other remedies such as adding the phosphodiesterase inhibitor (Cilostazol) or inhibiting Von Willebrand factor‐mediated thrombo‐inflammation, by blocking the platelet GPIbα binding site in the Von Willebrand factor A1 domain or by cleaving the Von Willebrand factor with ADAMTS13, can also affect blood flow and microvascular integrity.^90^ This highlights a method for NRP prevention in the future. Apart from the inhibition of platelets, early administration of alteplase, which targets fibrinogen‐dependent downstream microvascular thrombosis, may also optimize the effect of recanalization.^91^, ^92^, ^93^ One study found that intravenous thrombolysis pretreatment might increase the odds of successful reperfusion in patients undergoing endovascular thrombectomy.^94^ Recently, a phase 2b randomized clinical trial preliminarily indicated that an auxiliary therapy of intra‐arterial alteplase following recanalization (eTICI 2b50 or above) through endovascular thrombectomy appears to have a greater probability of excellent neurological outcome at 90 days in patients with successful reperfusion via thrombectomy.^95^

However, in published trials, controversial results still exist over the safety and efficacy of antiplatelet or thrombolytic therapy adjunct to reperfusion therapy, partially due to the heterogeneity in sample size, medication modality, therapeutic regimen, and trial protocol. The increased risk of intracranial hemorrhage transformation should also be carefully considered in future clinical trials.^96^

### 5.4 Vasodilators

Therapeutic strategies that prevent the sustained constriction of pericytes or swelling of the end‐feet in astrocytes are also considered promising for NRP management, as they may help to reduce the cerebral capillary resistance with subtle influence on the systematic circulation.^97^ Persistent contraction of pericytes could be reversed by the inhibition of oxidative‐nitrative stress and suppression of calcium influx.^19^, ^98^ In addition, adenosine and nitric oxide, with vasodilation, anti‐inflammation, and anti‐thrombus properties, have been demonstrated to restore microcirculation patency and improve reperfusion status, contributing to the overall survival of ischemic cerebral tissue during the ischemia–reperfusion process.^99^, ^100^ Considering the shared pathogenesis of the NRP in the heart and brain, other vasodilators (like nicardipine, nitroprusside, verapamil, and fasudil), that appear to reduce microvascular obstruction following PCI, also have the potential to ameliorate the NRP in stroke patients, though more studies must be carried out in both experimental models and stroke patients.^101^, ^102^

### 5.5 Ischemic conditioning

Ischemic conditioning, which refers to repetitive short episodes of ischemia with intermittent reperfusion, involves a complex network of molecular triggering and signaling pathways and has shown protective effects in both cardiovascular and cerebral vascular diseases.^103^ It can be divided into pre‐, per‐, and postconditioning according to the intervention timepoint, or termed as remote ischemic conditioning when applied to an organ away from the protected one.^104^ Preclinical studies have suggested its critical role in microcirculation modulation after reperfusion.^105^ Small sample trials have demonstrated that ischemic postconditioning attenuates no‐reflow extent in patients with acute myocardial infarction.^101,102^ More recently, an ongoing phase‐I study (NCT05153655) has been carried out to ascertain the safety and tolerability of ischemic postconditioning in stroke patients receiving mechanical thrombectomy. Future research regarding the efficacy of ischemic conditioning in the management of NRP should be explored.

### 5.6 Therapeutic hypothermia

Induced cooling, especially selective therapeutic cooling, is a promising technique for reducing the final infarct volume and improving outcomes in ischemic stroke.^106^, ^107^ It has been demonstrated to protect against the NRP in experimental models of acute myocardial infarction.^108^, ^109^ More compelling evidence is needed to support the efficacy of hypothermia in clinical settings and its correlation with cerebral reperfusion status after recanalization.^110^

## 6 FUTURE PERSPECTIVE

By now, advances in coronary NRP research have developed vastly and improved the outcomes of STEMI patients following PCI. Therefore, it is essential to take a page from the experience in coronary studies. In this section, we compared the similarities and differences between the coronary and cerebral NRP and discussed future directions for cerebral NRP investigations.

The main pathogenesis of the NRP in both the heart and brain lies in functional and structural damage to the microvasculature after prolonged cessation of artery occlusion and delayed restoration of blood flow. However, it is of note that cerebral blood flow regulation is more complicated compared with other circulation systems, with specific vascular structures, interaction between multiple cell types, and varieties of regulatory mechanisms and responses.^111^ Meanwhile, there is still no animal model available to mimic the pathophysiological process of cerebral NRP, which presents challenges for preclinical study.

In patients with acute coronary syndrome, there are abundant imaging modalities to identify and quantify the NRP including coronary angiography, cardiac magnetic resonance (CMR), myocardial contrast echocardiography (MCE), nuclear approaches, catheter‐based coronary physiology measurements, and electrocardiography, with measurements like TIMI flow, TIMI frame count, myocardial blush grade, ST‐segment resolution, index of microcirculatory resistance (IMR).^112^ CMR is currently considered the gold standard for coronary NRP assessment, based on which, a dark hypointense core in the areas of hyperenhancement that is identified early (~1 min) or late (15 min) after gadolinium injection is indicative of early or late CMVO, respectively.^113^ Certain risk factors have been detected in previous studies, including patient clinical characteristics, procedural variables, duration of ischemia, infarct location and size, and culprit plaque morphology. Modifiable risk factor control like intensive statin therapy and optimization of blood pressure and blood sugar has been used for coronary NRP prevention. In addition, process optimization to shorten onset‐to recanalization time, procedure improvement, intra‐coronary vasodilators, anti‐coagulant agents, anti‐platelet agents, and therapeutic hypothermia are now trialed in patients for the management of coronary NRP.^114^

However, our knowledge of the risk factors, prevention, and management of cerebral NRP in stroke patients is still scanty and conflicting due to the absence of consensus on detecting techniques, indicators, scales, and threshold value for post‐thrombectomy hypoperfusion identification and quantification. Thus, the top priority of future study is to establish a standardized definition and assessment criteria for the cerebral no‐reflow phenomenon. What follows is the investigation of risk factors of the cerebral NRP to identify patients at risk before thrombectomy and raise awareness for timely interventions, which may reduce the prevalence and extent of the NRP. Moreover, potential management derived from preclinical studies and coronary NRP experience should be trialed in stroke patients to evaluate the safety, feasibility, and efficacy and translated into a clinical effect on no‐reflow. In all, the future study of the cerebral NRP should mainly focus on pathophysiology, potential therapeutic targets, risk factors, and management translation. An integrated therapeutical management with timely identification, prevention, and multi‐target treatment seems promising to further improve the prognosis of stroke patients.

## 7 CONCLUSION

Microvascular obstruction after recanalization, which clinically manifests as reperfusion deficiency, namely the no‐reflow phenomenon, is common among ischemic stroke patients receiving thrombolysis or thrombectomy. Due to the significance of the reperfusion status for AIS patients, priority should be given to the in‐depth understanding of the pathogenesis, diagnosis, prevention, and management of the NRP to improve the prognosis of stroke patients receiving recanalization therapy.

## FUNDING INFORMATION

This study was supported by Beijing Natural Science Foundation (JQ22020), Beijing Nova Program (No. Z201100006820143), and the National Natural Science Foundation of China (No. 82001257, 81801313, and 81971114).

## CONFLICT OF INTEREST STATEMENT

The authors declare that they have no conflict of interest.

## Data Availability

Data sharing is not applicable to this article as no new data were created or analyzed in this study.
